# D471G Mutation in LCMV-NP Affects its Ability to Self-associate and Results in a Dominant Negative Effect in Viral RNA Synthesis

**DOI:** 10.3390/v4102137

**Published:** 2012-10-16

**Authors:** Emilio Ortiz-Riaño, Benson Y. H. Cheng, Juan C. de la Torre, Luis Martínez-Sobrido

**Affiliations:** 1 Department of Microbiology and Immunology, University of Rochester, 601 Elmwood Avenue, Rochester, New York 14642; Email: eortizriano@urmc.rochester.edu (E.O-R); benson_cheng@urmc.rochester.edu (B.Y.H.C); luis_martinez@urmc.rochester.edu (L. M-S); 2 Department of Immunology and Microbial Science, The Scripps Research Institute, La Jolla, California 92037; Email: juanct@scripps.edu (J.C. dlT)

**Keywords:** Lymphocytic choriomeningitis virus, nucleoprotein, Z matrix protein, self-association, viral-like particles, minigenome, type I Interferon, double-stranded RNA, dominant negative

## Abstract

Arenaviruses merit significant interest because several family members are etiological agents of severe hemorrhagic fevers, representing a major burden to public health. Currently, there are no FDA-licensed vaccines against arenaviruses and the only available antiviral therapy is limited to the use of ribavirin that is partially effective. Arenavirus nucleoprotein (NP) is found associated with the genomic RNA forming the viral ribonucleoproteins (vRNPs) that together with the polymerase (L) direct viral replication and transcription. Virion formation requires the recruitment of vRNPs into budding sites, a process in which the arenavirus matrix-like protein (Z) plays a major role. Therefore, proper NP-NP and NP-Z interactions are required for the generation of infectious progeny. In this work we demonstrate the role of the amino acid residue D471 in the self-association of lymphocytic choriomeningitis virus nucleoprotein (LCMV-NP). Amino acid substitutions at this position abrogate NP oligomerization, affecting its ability to mediate replication and transcription of a minigenome reporter plasmid. However, its ability to interact with the Z protein, counteract the cellular interferon response and bind to dsRNA analogs was retained. Additionally, we also document the dominant negative effect of D471G mutation on viral infection, suggesting that NP self-association is an excellent target for the development of new antivirals against arenaviruses.

## 1. Introduction

Arenaviruses cause rodent infections with a worldwide distribution [1]. Infections of humans are common and can occur by direct contact between infectious materials and abraded skin or inhalation of aerosol forms of the virus [1]. Arenaviruses are divided, according to serologic, genomic and geographic distribution, into two groups: Old World (OW) and New World (NW) arenaviruses [2]. Members of both groups can cause hemorrhagic fever (HF) disease in humans and pose a serious public health problem in their endemic regions [3,4]. The OW Lassa virus (LASV), the causative agent of Lassa fever (LF), is the HF arenavirus of highest impact in public health, with some 300,000 infections estimated yearly, which are associated with high morbidity and significant case fatality rates [5,6]. Moreover, increased travel to and from endemic regions has resulted in the importation of LF cases into non-endemic regions [7]. In addition, evidence indicates that the globally distributed prototypic arenavirus lymphocytic choriomeningitis virus (LCMV) is likely a neglected human pathogen associated with congenital infection leading to hydrocephalus, mental retardation and chorioretinitis in infants [8,9]. LCMV also poses a threat to immuno-compromised individuals, as shown by reported lethal cases of LCMV infection after organ transplantation [10,11]. Public health concerns posed by human pathogenic arenaviruses are aggravated by the lack of Food and Drug Administration (FDA)-licensed vaccines and current antiarenaviral therapy limited to off-label use of the nucleoside analog ribavirin, which is only partially effective and associated with significant side effects [12,13,14]. Additionally, several arenaviruses have been classified as National Institute of Allergy and Infectious Disease Category A Priority Pathogens because of their potential misuse as agents of bioterrorism [5]. Therefore, the development of novel effective antiviral strategies to combat human pathogenic arenaviruses are urgently needed, a task that would be facilitated by a better understanding of the arenavirus molecular and cell biology. On the other hand, LCMV has proven very useful as an experimental model system in the fields of viral immunology and pathogenesis [15,16].

Arenaviruses are enveloped, negative-sense RNA viruses with bisegmented genomes [1]. Each segment uses an ambisense coding strategy to direct the synthesis of two proteins in opposite orientation [17]. The Large (L) segment encodes for the RNA-dependent RNA Polymerase (L) and a small RING finger protein (Z) that has matrix-like functions [18,19], including directing the budding process [20]. The Small (S) segment encodes the glycoprotein precursor (GPC) and the nucleoprotein (NP). GPC is posttranslationally processed by the cellular site 1 protease to produce GP-1 and GP-2 [21,22], which associate to form the glycoprotein complex (GP) that constitute the spikes on the surface of the virion structure responsible for receptor recognition and cell entry [1]. NP encapsidates the viral genome, and together with the L protein, are the minimal components required for viral transcription and replication [23,24].

Recent publications have underscored the variety of roles played by NP during infection. Besides its involvement in virus replication and transcription, NP counteracts the host type I interferon (IFN-I) response by preventing activation of the interferon regulatory factor 3 (IRF3) and subsequent induction of IFN-I production and interferon-stimulated genes (ISGs) [25,26]. NP-mediated inhibition of IRF3 activation has been shown to correlate with NP’s ability to interact with IKKε, thus preventing phosphorylation of IRF3 [27]. The anti-IFN-I function of NP has been mapped to its C-terminal region that contains also a functional 3′-5′ exoribonuclease domain, believed to be responsible for the anti-IFN-I properties of NP [28,29,30]. Furthermore, the same domain and amino acid residues shown to play a critical role in NP anti-IFN-I activity have been recently shown to also mediate inhibition of nuclear translocation and transcriptional activity of Nuclear Factor Kappa B (NF-κB) [31]. These results suggest that arenaviruses may use a common mechanism to evade the host IFN-I and inflammatory responses, which likely plays a key role in arenavirus pathogenesis and virulence [32].

Mutation-function studies have also mapped the NP domains involved in NP self-association and NP-Z interaction at the N- and C- terminal regions, respectively [33,34,35,36]. Interestingly, the crystal structure of NP revealed the presence of head-to-tail NP-NP interactions, suggesting the potential role of both, the N- and the C-terminal regions of NP in self-association [29,37]. However, the contribution of specific residues to NP self-association has not been determined.

In this work, we provide evidence that an aspartic residue (D) at position 471 in LCMV-NP affects NP self-association and its ability, together with L, to replicate an LCMV minigenome (MG). However, mutations at D471 did not affect NP-Z interaction, NP binding to double-stranded (ds)RNA analogs or NP’s ability to counteract the cellular IFN-I antiviral response. Moreover, we also show that LCMV-NP with mutation D471G acts as a dominant-negative mutant that results in decreased viral replication in cell culture. Together, our findings suggest that NP-NP interaction might be an excellent target candidate for the development of new antivirals for the treatment of arenavirus infections.

## 2. Results

### 2.1. Identification of a Specific Amino Acid Residue with Critical Role in LCMV-NP Self-association

**Figure 1:**
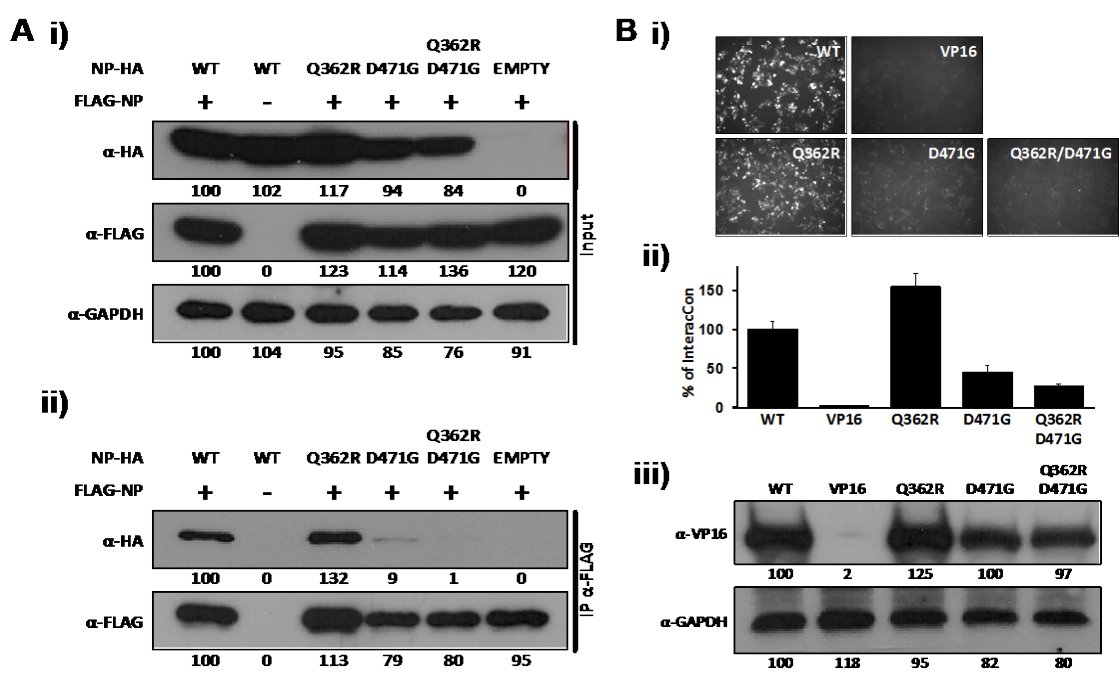
LCMV-NP D471G single amino acid mutant is affected in self-association. (A) Co-IP: Human 293T cells were co-transfected with 2 μg of the pC- LCMV-NP wt, single (Q362R and D471G), or double (Q362R/D471G) amino acid mutants HA-tagged, together with 2 μg of pC-LCMV-NP wt FLAG-tagged expression plasmid. As controls, LCMV-NP tagged versions were expressed individually together with 2 μg of empty pC to keep constant the total amount of transfected DNA. At 48 hpt, cell lysates were prepared and analyzed for protein expression levels by WB using anti-HA or anti-FLAG polyclonal antibodies (i). GAPDH was used as a loading control. Cell lysates were immunoprecipitated with anti-FLAG affinity agarose beads (ii) and analyzed by WB with the indicated antibodies (left). Numbers at the bottom of each WB lane represent the quantification of band intensities normalized to the signal of cells co-transfected with HA- and FLAG-tagged wt NPs, as described in Material and Methods. (B) M2H: Human 293T cells were co-transfected in triplicate with 2 μg of the indicated wt, single, or double amino acid mutant pC-VP16-tagged expression plasmids, together with 2 μg of LCMV-NP-GAL4 expression plasmid, along with 1 μg of the pG5 GFP/FFL dual reporter plasmid, and 0.1 μg of the pRL SV40 expression plasmid to normalize transfection efficiencies. At 72 hpt, GFP expression was assessed using fluorescence microscopy and cell extracts were prepared to determine the strength of NP-NP interaction using the Promega dual-luciferase reporter assay and a Lumicount luminometer. As negative control, we transfected cells with pC-NP-GAL4 together with pC-VP16. Representative fields of transfected cells are illustrated (**i**). Reporter gene activation (FFL) is shown as percentage (%) of LCMV NP-NP wt interaction (pC-NP-VP16 and pC-NP-GAL4) after normalization of transfection efficiencies with the Renilla luciferase expression plasmid pRL SV40 (ii). Cell lysates were used to detect expression of LCMV-NP wt and mutants by WB using an anti-VP16 polyclonal antibody (iii). GAPDH was used as a loading control. Numbers at the bottom of each WB lane represent the quantification of band intensities normalized to wt NP, as described in material and methods.

The ability of NP to self-associate has been recently demonstrated by biochemical studies [34,35]. This NP oligomerization likely plays a major role in the encapsidation of viral RNA and in the formation, together with L, of vRNPs that direct both replication and transcription of the viral genome [23,24]. To identify single amino acid residues involved in self-association of LCMV-NP, we screened a previously documented battery of LCMV-NP mutants [28] for their self-association properties. Our initial screen identified a double mutant containing Q362R and D471G substitutions that showed impairment in NP-NP interaction, as determined by co-immunoprecipitation (Co-IP) (Figure 1A) and the mammalian two-hybrid (M2H) (Figure 1B) assays. To assess the individual contribution of Q362R and D471 to NP-NP interaction, we generated NP mutant forms containing only mutation D471G or Q362R and tested them in our Co-IP and M2H assays. For Co-IP assays, we co-transfected 293T cells with N-terminal FLAG-tagged wild-type (wt) LCMV-NP (FLAG-NP), together with C-terminal HA-tagged versions of the LCMV-NP single (Q362R and D471G) or double (Q362R/D471G) mutants. Wt HA-tagged LCMV-NP was used as a validated control [34]. At 72 hours post-transfection (hpt), cell lysates were prepared and analyzed by Western blot (WB). NP mutant Q362R was expressed at similar levels as wt NP, while D471G and D471G/Q362R NP mutants were expressed at slightly lower levels (Figure 1A, i). Cell lysates were incubated with anti-FLAG affinity agarose gel and immunoprecipitates analyzed by WB using anti-HA or anti-FLAG antibodies. Band intensities were quantified and normalized with respect to total NP expression. D471G, but not Q362R, amino acid substitution disrupted NP-NP interaction (Figure 1A, ii). To confirm and further characterize the effect of D471G substitution in NP-NP interaction, we used the M2H system (Figure 1B). For this, we co-transfected 293T cells with pCAGGs (pC) plasmids expressing wt or mutant NPs fused to the N-terminus of VP16 (pC-NP-VP16) [33], together with plasmids expressing wt NP fused to the N-terminal of GAL4 (pC-NP-GAL4) [33] and the reporter plasmid encoding a green fluorescent protein (GFP) and Firefly luciferase (FFL) fusion protein, pG5 GFP/FFL. To normalize transfection efficiencies we included the plasmid pRL SV40. We used pC-VP16 as a negative control to show specificity of the assay [33,34]. We detected NP self-association of wt and Q362R NPs as determined by both GFP (Figure 1B, i) and FFL (Figure 1B, ii) reporter gene expressions. In contrast, D471G and the double mutant Q362R/D471G were severely impaired in their ability to interact with wt LCMV-NP. All VP16 fusion constructs were expressed at similar levels as determined by WB using an anti-VP16 polyclonal antibody (Figure 1B, iii). These results uncovered a key role of residue D471 in NP-NP interaction.

### 2.2. Role of D471 in LCMV NP-Z Interaction

**Figure 2:**
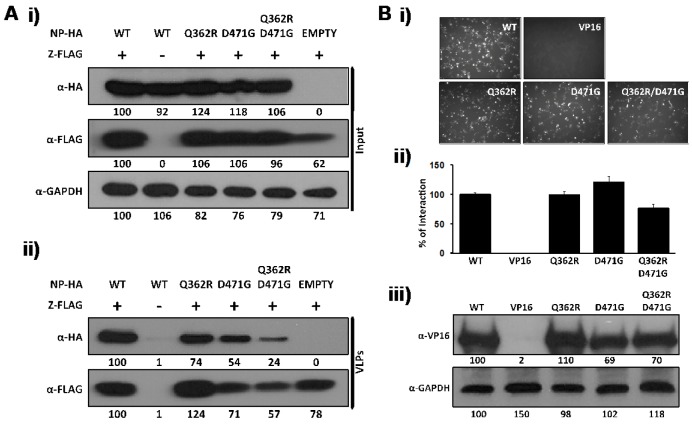
D471G amino acid substitution in LCMV-NP does not affect its interaction with LCMV-Z. (A) VLP assay: Human 293T cells were co-transfected with 2 μg of the indicated pC wt or mutant NPs HA-tagged, together with 2 μg of pC-LCMV-Z FLAG-tagged expression plasmid. Empty pC plasmid was included to normalize the total amount of transfected DNA. At 72 hpt, cell lysates were prepared and analyzed for protein expression levels for NP (α-HA) and Z (α-FLAG) (i). GAPDH was used as a loading control. TCS from same transfected cells were used for isolation of VLP to evaluate NP incorporation (α-HA) into Z-mediated VLP (α-FLAG) (ii). Numbers at the bottom of each WB lane represent the quantification of band intensities normalized to NP and Z wt expression levels. (B) M2H assay: Human 293T cells (6.5x10^5^) were co-transfected in triplicate (12-well plate format) with 2 μg of the indicated pC-VP16-tagged wt or mutant NPs, together with 2 μg of pC-GAL4-tagged Z expression plasmid as described in Figure 1B. At 72 hpt, LCMV NP-Z interaction was determined by GFP expression (i) and by luciferase activity (ii). pC-VP16 expression plasmid was used as negative control. Reporter gene activation (FFL) is shown as percentage of wt interaction (pC-NP-VP16 and pC-GAL4-Z) after normalization of transfection efficiencies with the Renilla luciferase expression plasmid pRL SV40. Expression levels of wt and mutant NP were determined by WB using an anti-VP16 polyclonal antibody (iii). GAPDH was used as a loading control. Numbers at the bottom of each WB lane represent the quantification of band intensities normalized to wt NP lane as described in material and methods.

Arenavirus Z protein has been shown to be the driving force of virus budding and in the absence of other viral proteins, mediates the formation of viral-like particles (VLP) [20,38,39,40]. Cell egress of arenavirus infectious progeny requires the interaction between Z and NP present in the vRNP. Accordingly, NP has been shown to be incorporated into Z-derived VLP [33,36,41]. The LCMV-NP domain involved in the interaction with Z was mapped to the C-terminal of viral NP [33]. To determine if residue D471 played a key role in the NP-Z interaction, we examined its interaction with Z using both VLP (Figure 2A) and M2H (Figure 2B) assays. For the VLP assay, a FLAG-tagged LCMV-Z pC expression plasmid was co-transfected in 293T cells alone or in combination with pC expression plasmids of HA-tagged wt or mutant NPs. At 72 hpt, tissue culture supernatants (TCS) and cell lysates were collected. WB analysis of cell lysates showed that all samples expressed similar levels LCMV-NP and LCMV-Z proteins (Figure 2A, i). VLP were purified through a 20 % sucrose cushion, and VLP-containing pellets were analyzed by WB [41]. All NP mutants tested were incorporated into the LCMV-Z-mediated VLP, although we observed a slight reduction for the Q362R/D471G NP mutant (Figure 2A, ii). As expected, NP was not detected in the TCS of 293T cells transfected only with pC-NP, demonstrating the specific NP incorporation into Z-derived VLP [33]. To further characterize the NP-Z interaction, we performed a M2H assay [33] (Figure 2B). In this case, 293T cells were co-transfected using pC plasmids expressing NP-VP16 fusion proteins containing wt or mutant forms of NP together with pC plasmids expressing GAL4-Z, the reporter plasmid pG5 GFP/FFL, and pRL SV40 to normalize transfection efficiencies. All mutants tested interacted at similar levels with Z as determined by GFP (Figure 2B, i) and FFL (Figure 2B, ii) reporter gene expression. All NP-VP16 fusion proteins were expressed at similar levels as determined by WB (Figure 2B, iii). Taken together, these results indicate that LCMV-NP residue D471 does not play a critical role in NP-Z interaction.

### 2.3. Role of NP Self-association in Virus RNA Replication and Gene Transcription

**Figure 3:**
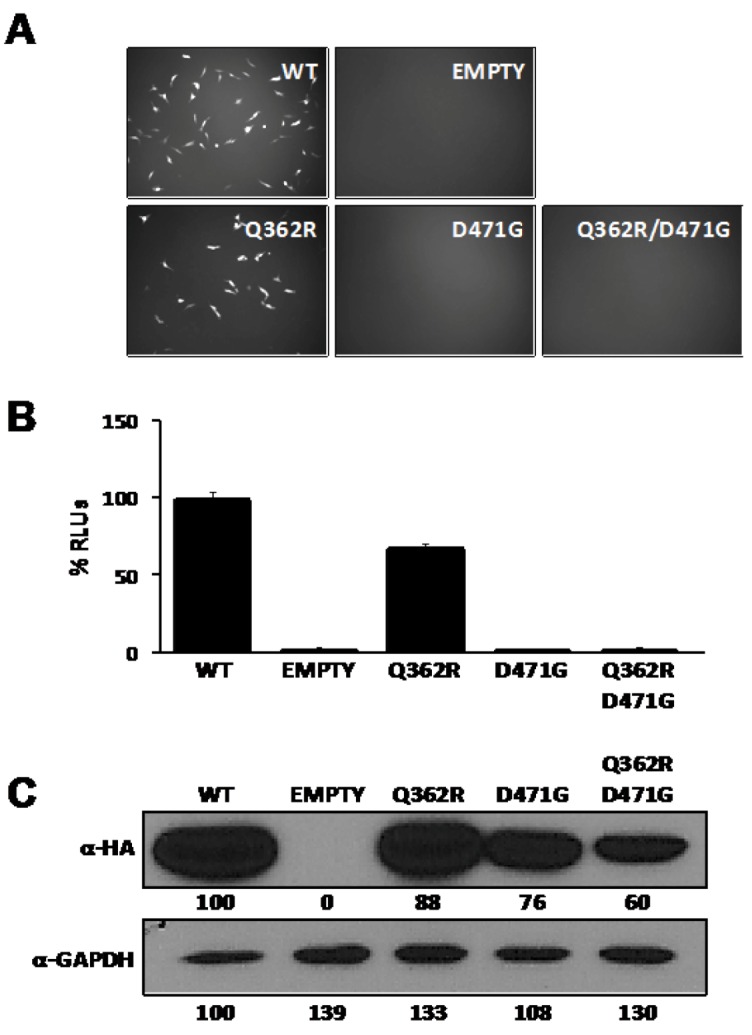
D471G substitution affects the ability of LCMV-NP to promote replication and gene expression of an LCMV MG. BHK-21 cells were co-transfected in triplicate with 0.5 μg of pPolI GFP-Pur/Gluc, together with 0.6 μg of pC-L, 0.3 μg of pC wt or mutant NPs HA-tagged, and 0.1μg of the pSV40-Cluc expression vector to normalize transfection efficiencies At 48 hpt, MG driven GFP expression (A) and luciferase activity in TCS (B) were determined. Cell lysates were used to determine expression levels of wt and mutant NPs by WB using an anti-HA antibody (C). GAPDH was used as a loading control. Empty pC was used as a negative control. Percentages of relative luciferase units (% RLUs) were normalized with respect to the activity of the wt NP, after normalization of transfection efficiencies based on Cluc luminescence values. Numbers at the bottom of each WB lane represent the quantification of band intensities normalized to wt NP lane as described in material and methods.

NP plays a critical role in arenavirus RNA replication and gene transcription [24,28], but whether monomers or multimers of NP are required to direct these RNA biosynthetic processes has not yet been determined. We therefore examined the activity of D471G NP mutant in an LCMV minigenome (MG) rescue assay [42]. In order to perform this task, we generated a dual reporter LCMV MG, where Pur-GFP and Gluc substituted for the LCMV-NP and -GPC open reading frames (ORFs), respectively, within the S segment that was flanked by the mouse pol-I promoter and terminator sequences (pPolI GFP-Pur/Gluc). BHK-21 cells were co-transfected with pPolI GFP-Pur/Gluc together with pC plasmids expressing L and HA-tagged versions of wt or mutant NPs and pSV40-Cluc to normalize transfection efficiencies. At 48 hpt, reporter gene expressions were assessed by fluorescence microscopy (Figure 3A) and luciferase activities from TCS (Figure 3B). Cells lysates were prepared and analyzed by WB to determine protein expression (Figure 3C). NP mutants containing the D471G substitution failed in their ability to replicate and transcribe the LCMV MG, while NP containing substitution Q362R showed a minimal defect on replication and transcription. Although we observed some variation in the expression levels among the different HA-tagged versions, the total absence of reporter gene expression associated with substitution D471G could not be attributed to the modest differences in protein expression levels observed by WB, indicating that NP-NP interaction is required for the function of NP in virus RNA replication and gene transcription.

### 2.4. Role of NP-self- association in the Anti-IFN-I Activity of NP

**Figure 4:**
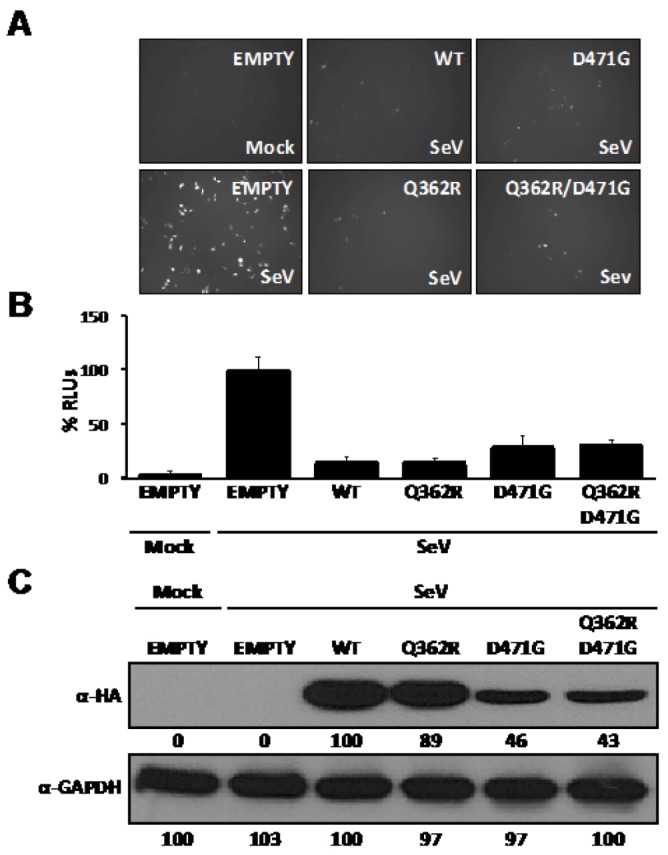
D471G amino acid substitution does not affect the anti-IFN-I function of LCMV-NP. Human 293T cells were co-transfected in triplicate with 0.5 μg of each of the IFNβ reporter plasmids (pIFNβ-GFP and pIFNβ-FFL), 0.1 μg of the indicated pC-NP HA-tagged expression vector, and 0.1 μg of pRL SV40 expression plasmid to normalize transfection efficiencies. At 16 hpt, cells were mock infected or infected with SeV (moi=3) to induce activation of the IFNβ promoter, and 24 hours later, GFP expression was assessed by fluorescence microscopy (A). Cell lysates were prepared for luciferase activities (B), and to detect expression of wt and mutant NPs by WB using an anti-HA antibody (C). GAPDH was used as a loading control. Reporter gene activation is shown as % of RLUs of a SeV-infected, empty-vector transfected control, after normalization by RL luminescence values. Numbers at the bottom of each WB lane represent the quantification of band intensities normalized to wt NP lane, as described in material and methods.

The anti-IFN-I activity of arenavirus NP [26] was mapped to the C-terminal region of the viral protein [28], whereas NP self-association was mapped to an N-terminal region of NP that did not overlap with the anti-IFN-I domain, suggesting that monomeric forms of NP can inhibit induction of IFN-I [34]. Since D471 plays a critical role in NP-NP interaction and is located within the C-terminal region of LCMV-NP responsible for the anti-IFN-I activity of NP, we examined whether substitution D471G that disrupted NP-NP association affected also the anti-IFN-I activity of NP (Figure 4). To that end, we co-transfected 293T cells with pIFNβ-GFP and pIFNβ-FFL [25] reporter plasmids and the indicated pC-NP HA-tagged expression plasmids, together with the pRL SV40, to normalize transfection efficiencies. At 16 hpt cells were infected (moi =3) with Sendai virus (SeV), [25] and at 24 hours post-infection (hpi), reporter gene expression was determined by fluorescence microscopy (Figure 4A) and luminescence (Figure 4B) assays. Both D471G and Q362R/D471G NP mutants were expressed to slightly lower levels than wt and Q362R mutant NPs as determined by WB (Figure 4C), but wt and all NP mutants tested similarly counteracted SeV-mediated induction of IFNβ, confirming that self-association of LCMV-NP is not required for counteracting the IFN-I response [34].

### 2.5. Characterization of Double-stranded (ds)RNA Binding Properties of LCMV-NP

**Figure 5:**
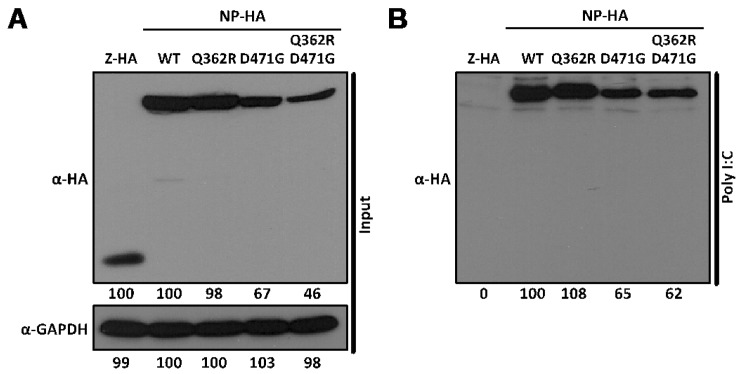
D471G amino acid substitution does not affect dsRNA binding of LCMV-NP. Human 293T cells were co-transfected with 2.5 μg of the indicated pC-LCMV-NP wt or amino acid mutants HA-tagged. pC-LCMV-Z HA-tagged was used as negative control for binding. At 48 hpt, cell lysates were prepared and analyzed for NP expression levels by WB using an anti-HA antibody (A). GAPDH was used as a loading control. Cell lysates were used in pull-down assays with poly I:C bound to sepharose beads and immunoprecipitates analyzed by WB (B). Numbers at the bottom of each WB lane represent the quantification of band intensities normalized to wt NP lane, as described in material and methods.

We have previously shown that self-association of LCMV-NP is mediated by RNA [34]. Furthermore, we have recently suggested the possibility that two different types of interactions mediate NP-NP self-association [34]. One interaction, requiring the participation of RNA, involves mainly a region located at the N-terminal region of NP [34]. The other interaction consists of an RNA-independent head-to-tail protein-protein interaction involving both the N- and C-terminal regions of NP [29,37]. This model would predict that NP-NP interaction mediated by the C-terminal domain of NP could be separated from the ability of NP to bind RNA. To test this, we evaluated the binding of wt and mutant NPs to the dsRNA analog poly I:C (Figure 5). As a control, we included LCMV-Z, which was predicted to not bind RNA. For this, we co-transfected 293T cells with the indicated pC-NP or pC-Z HA-tagged expression plasmids and 48 hpt, cells lysates were prepared and analyzed by WB (Figure 5A). Cell lysates were then incubated overnight with poly I:C sepharose beads. As an additional internal control and to demonstrate the specificity of NP-dsRNA binding, cell extracts were incubated with sepharose beads lacking poly I:C (data not shown). The beads were then washed and pull-down proteins were analyzed by WB. All LCMV-NPs, but not LCMV-Z, were pulled down in the presence of poly I:C sepharose beads (Figure 5B). As expected, none of the proteins tested were detected in the pull-down lacking polyI:C (data not shown). These results demonstrate that the LCMV-NP D471G mutation does not affect its ds RNA binding capability, and suggest that the NP self-association is not required for dsRNA binding.

### 2.6. Assessing the Functional Impact of Other Single Amino Acid Substitutions at Position D471 in LCMV-NP

**Figure 6:**
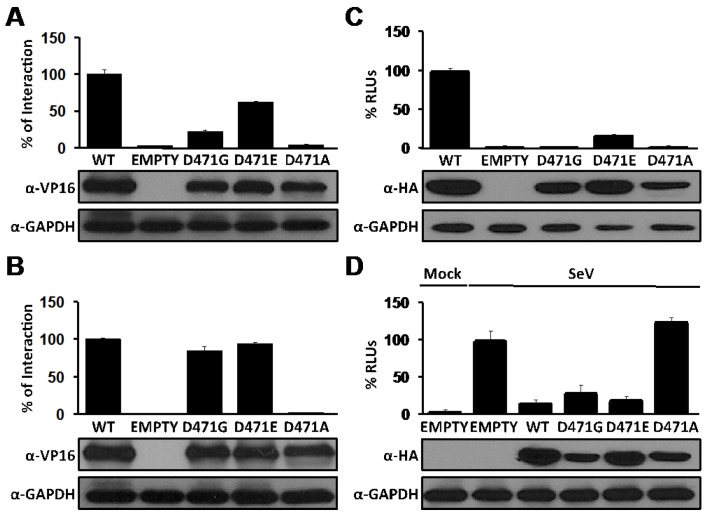
Effects of D471 substitution on LCMV-NP functions. (A) NP-NP interaction: Human 293T cells were co-transfected as described in Figure 1B with 2 μg of the indicated pC wt or mutant NP-VP16 fusion proteins, together with 2 μg of NP-GAL4 expression plasmids. At 72 hpt, cell extracts were prepared to determine the strength of the interaction. VP16 expression plasmid was used as negative control. Reporter gene activation (FFL) is shown as percentage of wt interaction (pC-NP-VP16 and pC-NP-GAL4) after normalization of transfection efficiencies based on levels of Renilla luciferase activity driven by plasmid pRL SV40. Cell lysates were used to detect expression of wt and mutant NPs by WB using an anti-VP16 polyclonal antibody. GAPDH was used as a loading control. (B) NP-Z interaction: Human 293T cells were co-transfected as described in Figure 2B with 2 μg of the indicated pC wt or mutant NP-VP16, together with 2 μg of GAL4-Z expression plasmids. At 48 hpt, cell extracts were prepared to determine the strength of the interaction and protein expression. Reporter gene activation (FFL) is shown as percentage of wt interaction (pC-NP-VP16 and pC-GAL4-Z) after normalization of transfection efficiencies based on Renilla luciferase values. Cell lysates were used to detect expression of wt and mutant NPs by WB using an anti-VP16 polyclonal antibody. GAPDH was used as a loading control. (C) Replication and transcription activity: BHK-21 cells were co-transfected with the LCMV MG as described in Figure 3 together with expression plasmids for the viral polymerase (L) and wt or indicated mutant NPs, and pSV40-Cluc expression vector to normalize transfection efficiencies. At 48 hpt, TCS were collected for luciferase assay and cell lysates were prepared for protein detection. Empty pC was used as a negative control. RLUs (%) were calculated based on the replication and transcription activity mediated by wt NP, after normalization by Cluc luminescence values. Expression levels of wt and mutant NPs were determined by WB using an anti-HA antibody. GAPDH was used as a loading control. (D) Inhibition of induction of IFN-I: Human 293T cells were co-transfected as described in Figure 4 with 0.1 μg of the indicated pC-NP HA-tagged expression vectors together with the IFNβ reporter plasmids. At 16 hpt, cells were infected with SeV (moi=3) to induce activation of the IFNβ promoter, and 24 hours later cell lysates were prepared for luciferase assay and detection of protein expression. Luciferase values were normalized with respect to those obtained in cells transfected with Empty pC and infected with SeV, after adjusting for renilla luminescence values. Expression of wt and mutant NPs were determined by WB using an anti-HA antibody. GAPDH was used as a loading control.

To further characterize the key role of residue D471 in the NP-NP interaction and RNA synthesis, but not in NP-Z interaction or anti IFN-I activity, we introduced additional amino acid substitutions at position 471 (D471E and D471A) and evaluated their contribution to NP-NP and NP-Z interactions, as well as their role in replication and expression of the LCMV MG and anti-IFN-I activity of NP (Figure 6). The conservative change D471E resulted in reduced levels of NP self-association (Figure 6A) without affecting NP-Z interaction (Figure 6B). Interestingly, the D471E substitution allowed replication and transcription of the LCMV MG, although to lower levels compared to wt NP (Figure 6C). Likewise, D471E was not affected in its ability to inhibit SeV-mediated induction of IFN-I (Figure 6D). Unexpectedly, the D471A substitution impeded both NP-NP and NP-Z interactions, as well as NP functions in replication and transcription of the LCMV MG and the ability of NP to counteract SeV-mediated induction of the host IFN-I response, suggesting that change D to A at position 471 might have affected the overall structure of NP without affecting its stability as reflected by its unchanged expression levels determined by WB using anti-VP16 (Figure 6A and 6B) or anti-HA (Figure 6C and 6D) antibodies.

### 2.7. Assessing the Dominant Negative Phenotype of LCMV-NP D471G

**Figure 7:**
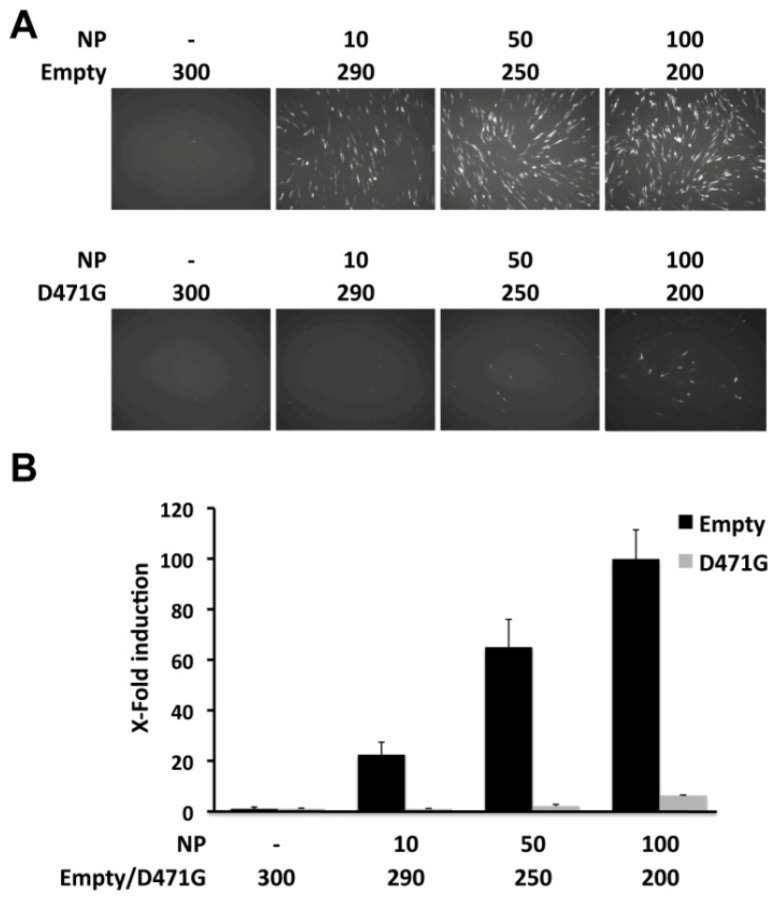
Dominant negative effect of LCMV-NP D471G on viral replication and transcription. BHK-21 cells were co-transfected in triplicate with 0.5 μg of pPolI GFP-Pur/Gluc, 0.6 μg of pC-L, and the indicated amounts (ng) of pC-LCMV-NP HA-tagged (NP); together with empty pC (Empty) or pC LCMV-NP D471G HA-tagged (D471G), and 0.1μg of the pSV40-Cluc expression vector to normalize transfection efficiencies. At 48 hpt, MG activity was determined by GFP expression (A) and luciferase activity from TCS (B). Reporter gene activation is shown as induction over an empty pC vector-transfected control.

Consistent with its lack of self-association, LCMV-NP D471G was not able to promote replication and expression of the LCMV MG. However, based on the dsRNA-binding assay, NP D471G may potentially keep its interaction with the viral RNA, thus possibly interfering with viral replication and transcription by blocking the viral polymerase extension on the RNA or its interaction with L. This would predict a dominant negative phenotype of NP D471G in the LCMV MG rescue assay. To examine this possibility, BHK-21 cells were co-transfected with either empty pC or pC NP D471G together with our MG reporter plasmid pPolI GFP-Pur/Gluc, along with pC L and increasing amounts of pC wt NP-HA to establish a linear response range. Transfection efficiencies were normalized using pSV40-Cluc. At 48 hpt, reporter gene expressions were assessed by fluorescence microscopy (Figure 7A) and luciferase activities from TCS (Figure 7B). Transfected cells containing LCMV-NP D471G mutant showed strong reduction in the replication and transcription of the LCMV MG at the different concentration tested compared to cells transfected with empty plasmid. These results suggest a dominant negative effect of D471G mutant on the MG assay, possibly by interfering with the replication and transcription function.

**Figure 8:**
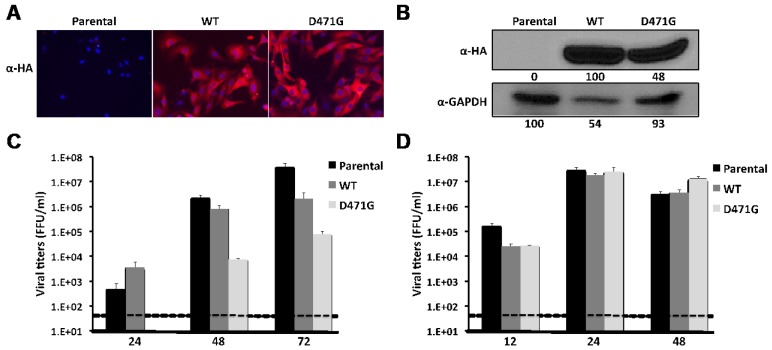
Dominant negative effect of NP D471G on LCMV infection. (A) Characterization of stable cell lines expressing HA-tagged wt mutant or D471G NPs. Parental and NP-HA expressing BHK-21 cell lines were examined by immunofluorescence with an anti-HA (α-HA) antibody (NP staining). Cellular nuclei were stained with DAPI. Representative merged images are illustrated. Cells lysates were prepared and 100 μg of total cellular protein content were analyzed by WB using an anti-HA antibody (B). GAPDH was used as a loading control. Numbers at the bottom of each WB lane represent the quantification of band intensities normalized to wt NP lane, as described in material and methods. Kinetics of LCMV (C) and VSV-GFP (D) propagation: Parental and NP-HA expressing BHK-21 cell lines were infected with LCMV (moi = 0.01) or VSV-GFP (moi = 0.001) in triplicates. TCS at the indicated hpi were titrated using a focus forming unit assay on Vero cells as described in materials and methods. Dashed lines indicate the limit of detection for the assay.

The observed dominant negative effect on the replication and transcription in the MG assay led us to hypothesize that mutation D471G in LCMV-NP would also interfere with viral infection. Supporting this hypothesis, LCMV-NP D471G mutant might still interact with the Z protein, therefore interfering with the Z-vRNP interaction required for formation of matured infectious progeny [33,35]. To test this, we generated BHK-21 stable cell lines expressing HA-tagged versions of wt or D471G NPs (Figure 8A, B) and examined their susceptibility to LCMV in comparison to parental BHK-21 cells. For this, we infected the different cell lines at low moi (0.01) with LCMV and determined levels of infectious progeny in TCS at 24, 48 and 72 hpi (Figure 8C). Cells expressing D471G mutant displayed reduced viral titers at all time points tested. Intriguingly, at 24 hpi virus titers in TCS were higher in cells expressing wt NP than in BHK-21 parental, but peak titers in cells expressing wt NP did not reach the titers obtained in parental BHK-21 cells, suggesting that over-expression of LCMV-NP might interfere with optimal production of infectious progeny. This dominant negative effect, however, was not observed in cells infected with a Vesicular Stomatitis Virus expressing GFP (VSV-GFP, Figure 8D), confirming that the stable cell line expressing D471G NP has a specific effect on LCMV viral proliferation. 

## 3. Experimental Section

### 3.1. Cells and Viruses

Baby hamster kidney (BHK-21) cells (ATCC CCL-10) and human embryonic kidney (293T) cells (ATCC CRL-11268) were maintained in Dulbecco’s Modified Eagle’s Medium (DMEM) supplemented with 10 % fetal bovine serum (FBS), L-glutamine (2mM), penicillin (100 units/ml) and streptomycin (100μg/ml), in a 5 % CO_2_ atmosphere at 37°C [25,43]. LCMV (Armstrong strain, ARM53b) was produced by infecting BHK-21 cells (moi = 0.01) and harvesting the tissue culture supernatants (TCS) at 72 hours post-infection (hpi) as previously described [43]. Vesicular stomatitis virus expressing GFP (VSV-GFP) was produced by infecting BHK-21 cells (moi = 0.001) and harvesting the TCS at 48 hpi [44].

### 3.2. Plasmids

Hemagglutinin (HA)- and FLAG-tagged versions of LCMV-NP and -Z in pCAGGs (pC) [26,33], as well as LCMV-NP and -Z open reading frames (ORFs) fused to VP16 and GAL4 in pC [33,34], have been described. LCMV-NP single amino acid mutants (Q362R, D471G, D471E, and D471A) and the double mutant (Q362R and D471G) were generated by site-directed mutagenesis (Stratagene) and then sub-cloned into pC-HA and -VP16 expression plasmids to generate the corresponding C-terminal fusion proteins [27]. The pG5 FFL reporter plasmid (Promega) was modified fusing the Green Fluorescent Protein (GFP) ORF to the N-terminal region of Firefly luciferase (FFL) coding sequence to generate pG5 GFP/FFL [33].

For the MG reporter assays, a fusion construct of Puromycin (Pur) and Green Fluorescent Protein (GFP) was amplified by PCR from the p18Kprom-Puro-EGFP plasmid, kindly provided by Drs. G. Chen and J. Roberts [45], with primers containing BbsI restriction sites and cloned into the LCMV S segment backbone pPolI GPC/BbsI plasmid [46] to generate the pPolI GPC/Pur-GFP. Similarly, *Gaussia* Luciferase (Gluc) was amplified by PCR from the pC-Gluc plasmid [47] with primers containing BsmBI restriction sites and cloned into the LCMV S segment backbone pPolI BsmBI/NP plasmid [46] to generate the pPolI Gluc/NP. Both plasmids (pPolI GPC/Pur-GFP and pPolI Gluc/NP) were then combined using BglII restriction sites to generate the dual LCMV MG reporter plasmid pPolI Gluc/Pur-GFP, where the Pur-GFP fusion replaces the NP ORF and Gluc replaces the GPC ORF and the 5’-and 3’ non-coding untranslated regions (UTRs) of the LCMV S segment viral RNA [23] were flanked by the mouse polymerase I promoter and terminator sequences, respectively [48]. pIFNβ-GFP/CAT and pIFNβ-FFL, for the IFN-I induction assay, have been previously described [25].

Primers used to generate the described plasmids are available upon request. Constructs ORFs were verified by DNA sequencing and protein expression confirmed by Western blot (WB). 

### 3.3. Co-immunoprecipitation (Co-IP)

Human 293T (3 x 10^6^ cells) were co-transfected in suspension (6-well tissue culture plates format) with 4 μg total of HA- and FLAG-tagged NP pC expression plasmids using Lipofectamine 2000 (Invitrogen), according to manufacturer’s instructions [34]. Empty pC plasmid was included to maintain a constant total (4 µg) amount of transfected plasmid DNA. At 48 hours post-transfection (hpt), cells were collected by centrifugation at 1,000 rpm for 30 minutes (min) at 4°C and lysed with 400 μl of lysis buffer (20 mM Tris-HCl pH 7.4, 5 mM EDTA, 100 mM NaCl, 1% NP-40, complete cocktail of protease inhibitors, Roche) for 30 min on ice. Cell lysates were clarified by centrifugation at 1,000 rpm for 30 min at 4°C. Aliquots (20 µl; 5% of total cell lysate) were analyzed for protein expression by gel electrophoresis and WB. For immunoprecipitation, 50 μl of EZview Red anti-FLAG M2 affinity gels (Sigma) were pre-incubated for 10 min with lysis buffer at room temperature and used to immunoprecipitate 50 μl (12.5 % of total cell lysates) of cleared lysates overnight at 4 °C. Affinity gels were washed four times with 500 μl of lysis buffer and immunoprecipitated samples were resuspended in 50 μl of sodium dodecyl sulfate-polyacrylamide gel electrophoresis (SDS-PAGE) loading buffer. A 25 μl aliquot of each immunoprecipitated sample was analyzed by WB [34].

### 3.4. Virus-Like Particle (VLP) Assay

To generate VLPs, human 293T cells were co-transfected in suspension (6-well plate format, 3 x 10^6^ cells/well) with 2 μg of the pC-Z-FLAG expression plasmid and 2 μg of wild type (wt) or mutant LCMV-NP expression plasmids [33] using 1 μg of Lipofectamine 2000 per μg of DNA. Empty pC plasmid was used to keep constant the total amount of transfected plasmid DNA. At 72 hpt, cells and TCS were collected. TCS were clarified at 1,000 rpm for 30 min and then layered on top of a 20 % sucrose cushion and centrifuged at 35,000 rpm on an SW-41 rotor for 2.5 hours. VLP-containing pellets were resuspended in 100 μl of 1X phosphate-buffered saline (1X PBS), and 20 μl were analyzed by WB. Cell pellets were lysed with 400 μl of lysis buffer (10 mM Tris-HCl, pH 7.4, 5 mM EDTA, 100 mM NaCl, 1% NP-40, complete cocktail of protease inhibitors; Roche) for 30 min on ice. Cell lysates were clarified by centrifugation at 1,000 rpm for 30 min at 4 °C. Aliquots (20 μl; 5 % of total cell lysates) from each sample were analyzed by WB [33].

### 3.5. Minigenome (MG) Assay

BHK-21 cells (2 x 10^5^) were co-transfected in monolayer (12-well plate format, triplicates) with 0.6 μg of pC-L, 0.5 μg of the dual LCMV MG reporter plasmid pPolI Gluc/Pur-GFP, indicated amounts of pC-LCMV-NP HA-tagged, and 0.1 μg of a mammalian expression vector encoding the secreted *Cypridina* luciferase (Cluc) under the control of the Simian Virus 40 (SV40) constitutive promoter (pSV40-Cluc, New England Biolabs) to normalize transfection efficiencies, using 2.5 μg of Lipofectamine 2000 per well. At 48 hpt, Pur-GFP expression was assessed by fluorescence microscopy using a Zeiss fluorescent microscope and luciferase activities in TCS determined using a Lumicount luminometer (Packard). Representative images of GFP expression are shown. Gluc activity was determined using the dual luciferase reporter assay (Promega) and Cluc activity by the Biolux *Gaussia* kit (New England Biolabs). Reporter gene activation (Gluc) is indicated as a percentage of relative light units (% RLUs) of LCMV-NP wt or X-Fold induction over negative transfected-control, where pC-LCMV-NP HA-tagged expression plasmid was replaced by empty pC, after normalization with the Cluc luminescence values Mean value and standard deviation were calculated using Microsoft Excel software [33].

### 3.6. Mammalian Two-hybrid (M2H) Assay

Human 293T cells (6.5x10^5^) were co-transfected in suspension in 12-well tissue culture plates, using Lipofectamine 2000. Each well contained 2 μg of the indicated pC-VP16 and GAL4 expression plasmids, 1 μg of the reporter pG5 GFP/FFL plasmid, and 0.1 μg of a Renilla luciferase (RL) expression plasmid under the control of the SV40 promoter (pRL SV40, Promega) to normalize transfection efficiencies. At 72 hpt, representative fluorescence images were obtained to evaluate protein-protein interaction by GFP expression using a Zeiss fluorescent microscope. Upon imaging, cell lysates were prepared to determine luciferase activities using the Promega dual-luciferase reporter assay and a Lumicount luminometer (Packard). Percentage of interaction of LCMV-NP mutants with wt LCMV-NP or LCMV-Z, after normalization of transfection efficiencies with the RL expression plasmid pRL SV40, was calculated based on wt NP-NP interaction (pC-NP-VP16 and pC-NP-GAL4) and wt NP-Z interaction (pC-NP-VP16 and pC-GAL4-Z), respectively. M2H experiments were performed in triplicate. The mean and standard deviation were calculated using Microsoft Excel software. Protein expression was determined by WB as described [33]. Mean value and standard deviation were calculated using Microsoft Excel software [33].

### 3.7. Double-stranded (ds)RNA Pull Down Assay

For dsRNA pull down assays, human 293T cells (3 x 10^6^, 6-well plate format) were co-transfected in suspension, harvested, and cell lysates prepared as described for Co-IP assays. Aliquots (20 μl; 5 % of total cell lysates) were analyzed for protein expression by gel electrophoresis and WB. For dsRNA binding, Polyinosine-polycytidylic acid (poly I:C, Sigma) was bound to CNBr-activated sepharose 4B (GE Healthcare) following manufacturer’s recommendation. Poly I:C sepharose beads (50 μl) were pre-incubated for 10 min with lysis buffer at room temperature and used to pull-down 100 μl (25 % of total cell lysates) of cleared lysates overnight at 4 °C. Sepharose beads were washed four times with 500 μl of lysis buffer and pull-down samples were resuspended in 50 μl of SDS-PAGE loading buffer. A 25 μl aliquot of each pull-down sample was analyzed by WB. To control for specific binding, sepharose beads without poly I:C bound were included during the pull-down assay (data not shown).

### 3.8. Type I Interferon (IFN-I) Reporter Assay

Human 293T cells were co-transfected (2 x 10^5^ cells/well, 12-well plate format, triplicate) using a calcium phosphate method with 0.5 μg of each of the IFNβ reporter plasmids (pIFNβ-GFP/CAT and pIFNβ-FFL [25]) and 0.1 μg of the pC-LCMV-NP HA-tagged expression plasmids, together with 0.1 μg of pRL SV40 to normalize transfection efficiencies. After overnight transfection, cells were infected with Sendai virus (SeV, moi=3), and 24 hpi, fluorescence images were obtained to determine IFNβ promoter activation by GFP expression [25]. After imaging, cell lysates were prepared for luciferase activities and protein expression [33]. Luciferase activities were measured by using the dual-luciferase kit (Promega) as recommended by the manufacturer. Reporter gene activation is shown as % RLUs of the infected empty-plasmid-transfected control. Protein expression was determined by WB as previously described [33]. Mean value and standard deviation were calculated using Microsoft Excel software [33].

### 3.9. Protein Gel Electrophoresis and Western Blot (WB) Analysis

Proteins were separated by 7.5 % or 12 % SDS-PAGE and then transferred onto nitrocellulose membranes (Bio-Rad) overnight at 4 °C. After blocking for one hour at room temperature with 10 % dry milk in 1X PBS, membranes were incubated with polyclonal primary antibodies against HA and FLAG (Sigma H6908 and F7425, respectively), a polyclonal antibody against VP16 (Sigma V4388), or a monoclonal antibody against glyceraldehyde-3-phosphate dehydrogenase (GAPDH, AbCAM AB9484) for one hour at room temperature. Membranes were then washed three times with 1X PBS containing 0.1 % Tween-20, and probed with secondary horseradish peroxidase-conjugated anti-mouse or anti-rabbit immunoglobulin (Ig) antibodies (GE Healthcare UK) for one hour at room temperature. After three washes with 1X PBS containing 0.1 % Tween-20, proteins were detected using a chemiluminescence kit and autoradiography films from Denville Scientific Inc. Protein band intensities were quantified using imageJ software (National Institutes of Health, NIH). Band quantifications for inputs of Co-IP and VLP assays, M2H assay, MG assay, and IFN-I induction assay were normalized according to GAPDH expression and assigning 100 % intensity to wt LCMV-NP. LCMV-NP mutants expressions were then normalized by their relative intensity to wt LCMV-NP. 

### 3.10. Generation of NP-expressing Stable Cell Lines

BHK-21 cells constitutively expressing carboxy-terminal HA-tagged LCMV-NP wt or the D471G mutant were generated as previously described [43,49,50]. Briefly, BHK-21 cells were co-transfected with pC-LCMV-NP wt or D471G HA-tagged, together with the puromycin resistance vector pPUR (Clontech) (7:1 ratio) using Lipofectamine 2000. At 48 hpt, cells were plated into 10-cm dishes at low density and cell clones were selected in the presence of 2.5 μg/ml of puromycin (Cellgro, 61-385-RA). Puromycin resistant clones were screened for NP expression by immunofluorescence and WB using a polyclonal antibody against the HA epitope (Sigma H6908). The best LCMV-NP expressing BHK-21 clones were maintained in DMEM supplemented with 10 % FBS, penicillin/streptomycin, and 2.5 μg/ml of puromycin.

### 3.11. Virus Growth Kinetics and Titrations

Growth kinetic analyses of LCMV and VSV-GFP were performed in parental and NP-expressing BHK-21 cell lines (6-well plate format). Sub-confluent cell monolayers (2x10^5^ cells/well) were infected in triplicates with LCMV ARMS53b and VSV-GFP at a low moi (0.01 and 0.001, respectively). After 90 min of adsorption at 37 °C for LCMV and 60 min at room temperature for VSV-GFP, virus inoculum was removed, cell monolayers were washed twice with PBS, and maintained in post-infection media (1:1 mixture of Opti-MEM reduced serum medium and DMEM containing 10 % FBS for LCMV and DMEM containing 0.3 % of bovine albumin for VSV-GFP). At the indicated times post-infection, TCS were collected, clarified by centrifugation, and titrated. Virus titers (focus-forming units [FFU]/ml) in TCS were determined by immunofocus assay [51] for LCMV and by fluorescent focus assay for VSV-GFP. Briefly, for LCMV, Vero cells were infected with 10-fold serial dilutions of TCS for 90 min at 37 °C. At 24 hpi, cells were fixed with 4 % formaldehyde in 1X PBS for 15 min, permeabilized with 0.2 % Triton X-100 in 1X PBS for 10 min at room temperature, and blocked overnight with 2.5 % bovine serum albumin (BSA) in 1X PBS. LCMV infected cells were identified by staining with an anti-NP mAb (1.1.3) [43] and a 1:200 dilution in 2.5 % BSA of a fluorescein isothiocyanate (FITC)-conjugated rabbit anti-mouse IgG secondary antibody (Dako, F0261). LCMV-NP positive cells were counted by fluorescence microscopy. For VSV-GFP, Vero cells were infected with 10-fold serial dilutions of TCS for 60 min at room temperature. After viral absorption, cells were maintained in post-infection media. At 12 hpi, VSV-GFP infected cells were determined by GFP expression and counted under a fluorescent microscopy. Mean value and standard deviation were calculated using Microsoft Excel software [43].

## 4. Conclusions and Discussion

For a variety of negative strand RNA viruses, self-association of the virus nucleoprotein has been shown to be required for the formation of functional vRNP that directs replication and transcription of the viral genome [52,53,54,55,56]. In this work, we have strongly suggested the role of the residue D471 in the self-association of NP from the prototypic arenavirus LCMV. The substitution D to G at position 471 abrogated the self-association property of LCMV-NP, affecting its ability to promote replication and transcription of an LCMV MG. However, the D471G mutant was not affected in its ability to interact with Z, counteract the SeV induced IFN-I response or bind to dsRNA analogs. Amino acid substitution to a similarly charged E residue partially restored the properties lost by the D to G substitution, while D to A substitution disrupted all NP’s interactions and functions tested, probably by affecting the overall NP structure. In addition, we have documented the dominant negative effect of the D471G mutant on virus multiplication, suggesting that NP self-association is a potential target for the development of new therapeutics against arenaviruses.

**Figure 9:**
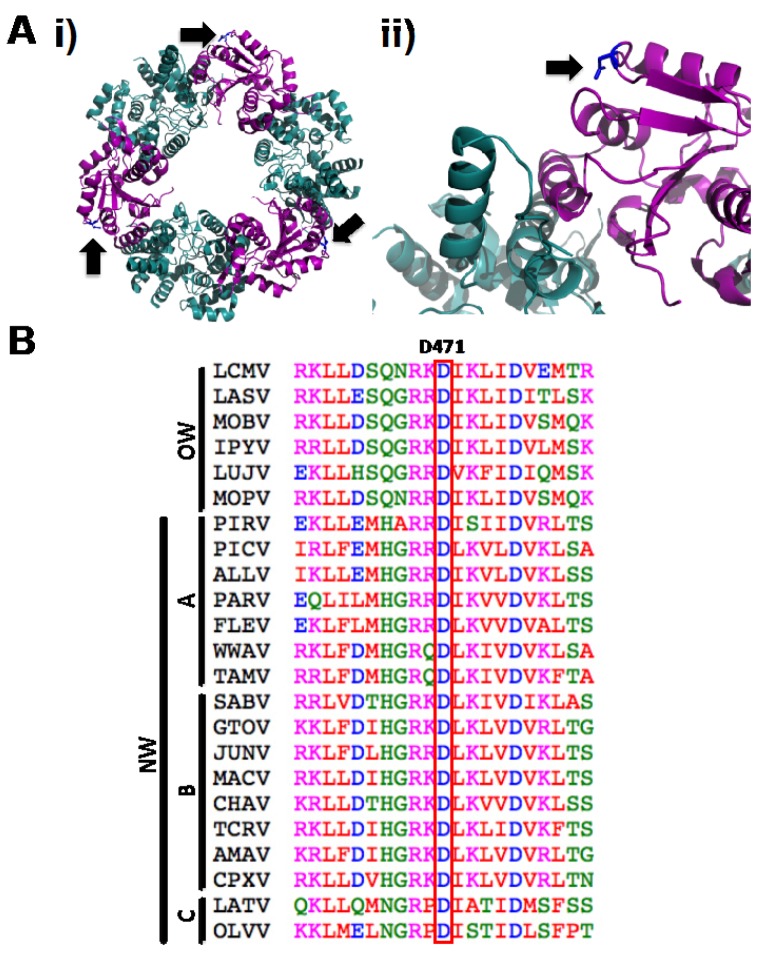
Structural extrapolation and sequence alignment analysis of LCMV-NP D471 residue. (A) Cartoon diagrams of LASV-NP crystal structure [29] indicating localization of amino acid residue D471. (i) Residue D471 in the trimeric structure, indicated by black arrows. (ii) N-C interface of the LASV-NP structure, where the side chain of residue D471 is indicated in blue and pointed by a black arrow. N-terminal and C-terminal regions of LASV-NP are indicated in cyan and magenta, respectively. (B) D471 amino acid residue is highly conserved between arenavirus NPs. Amino acid sequence alignment of arenavirus NPs of the region spanning D471 residue. Representative members of Old World (OW) and the New World (NW) clades A, B and C arenaviruses were included. LCMV, Lymphocytic choriomeningitis virus (AY847350.1); LASV, Lassa virus (HQ688673.1); MOBV, Mobala virus (AY342390.1); IPYV, Ippy virus (NC_007905.1); LUJV, Lujo virus (JX017360.1); MOPV, Mopeia virus (NC_006574.1); PIRV, Pirital virus (NC_005894); PICV, Pichinde virus (AF081553.1); ALLV, Allpahuayo virus (NC_010253.1); PARV, Parana virus (AF512829.1); FLEV, Flexal virus (NC_010757.1); WWAV, White water arroyo virus (EU486820.1); TAMV, Tamiami virus (EU486821.1); SABV, Sabia virus (NC_006317.1); GTOV, Guanarito virus (NC_005077.1); JUNV, Junin virus (JN801476.1); MACV, Machupo virus (NC_005078.1); CHAV, Chapare virus (NC_010562.1); TCRV, Tacaribe virus (NC_004293.1); AMAV, Amapari virus (NC_010247.1); CPXV, Cupixi virus (NC_010254.1), LATV, Latino virus (AF512830.1); OLVV, Oliveros virus (NC_010248.1). ClustalW2 program was used for alignment [57]. Amino acid are colored according to chemical properties: red (hydrophobic and aromatic amino acids), blue (acidic), magenta (basic), green (hydroxyl and amine containing), as specified by the EMBL-EBI CLUSTALW 2.0.8 multiple sequence alignment program.

We have shown that the mutation D471G in LCMV-NP disrupts its ability to self-associate by using Co-IP (Figure 1A) and M2H (Figure 1B) approaches. However, this amino acid mutation is localized in the C-terminal region of LCMV-NP, which is outside of the previously described domain required for NP oligomerization [34,35]. Analysis of the only available arenavirus NP crystal structure, that of LASV-NP [29] (Figure 9A) shows that the residue in LASV-NP corresponding to D471 in LCMV is not located in the proposed protein-protein interaction interface, but its side chain points towards the intramolecular N-C interface. NP sequence alignment from representative members of the *Arenaviridae* family shows the highly conserved nature of the D471 residue (Figure 9B), suggesting its structural or functional, or both, importance across the different arenaviruses. Interestingly, it has been suggested that LASV-NP has a possible gating mechanism that allows protein structural rearrangement upon RNA binding [37]. However, the C-terminal region organization has not been determined for this newly adopted conformation. Based on biochemical and structural analysis we proposed that the NP self-association might involve both the N-terminal and the C-terminal region. It is plausible that in the newly formed NP open conformation following RNA binding to the N-terminal domain of NP, residue D471 becomes engaged in NP oligomerization via direct protein-protein interaction.

However, we have previously shown that N-terminal deletion mutants of NP, which were defective in NP-NP interaction, retained the ability to counteract the IFN-I response [28,34]. Accordingly, in the present work, we have shown that the D471G mutation affected NP-NP interaction without disrupting the NP’s anti-IFN-I activity (Figure 4). Likewise, although the C-terminal region of LCMV-NP was implicated in NP-Z interaction [33], mutation D471G did not affect the ability of NP to interact with the Z protein in both VLP (Figure 2A) and M2H (Figure 2B) assays. Together, these findings strongly suggest the presence of domains with distinct functions within the C-terminal region of LCMV-NP. Moreover, results obtained with the D471G NP mutant further support the conclusion that monomers of NP are sufficient to counteract the host IFN-I response.

Previous studies documented that N-terminal deletions affecting NP-NP interaction abrogated NP’s ability to promote RNA replication and gene expression of an LCMV MG [28,34]. However, we did not have available a single amino acid mutant affecting NP self-association to corroborate its effect on the replication and transcription function. Confirming our hypothesis, the D471G mutant failed to replicate and transcribe a MG reporter plasmid (Figure 3), when co-transfected with the L protein. Supporting our data, mutations in amino acids predicted to be in the NP-NP interface have been described to also abrogate the replication and transfection function [37]. Taken together, these results suggest that proper oligomerization of NP is necessary for viral replication and transcription, where the overall structural protein integrity is required.

NP-mediated encapsidation of arenavirus genome and antigenome RNA species, which involves NP-RNA interaction, is strictly required for replication and gene expression of viral genome. Biochemical and structural data indicate that NP self-association involves a RNA-dependent protein-protein interaction mediated by the N-terminal region of NP [34]. In the absence of viral RNA, NP self-association can be mediated by cellular RNA [34]. The mechanisms whereby in virus-infected cells NP discriminates between viral from cellular RNA remain to be elucidated. Likewise, the structural features of the RNA substrate recognized by NP to mediate encapsidation have not been defined. However, as with other NS RNA viruses, arenaviruses exhibit sequence complementarity between the 5’-and 3’-termini of their genomes and antigenomes, which is predicted to result in the formation of a panhandle structure that could provide the structural scaffold for both the virus promoter and nucleation site for RNA encapsidation [58,59]. Our results have shown that D471G mutant NP, deficient in NP-NP interaction, was capable of binding to the dsRNA analog poly I:C (Figure 5), suggesting that the initial NP-RNA interaction is not required for the self-association of NP. The contribution of residue D471 to the multiple functions associated with NP was further supported by the finding that substitution D471A disrupted all tested NP interactions and functions (Figure 6). This likely reflected the limited rotational flexibility of A compared to G [60], which could have resulted in the disruption of the overall structure in D471A mutant NP.

The D471G mutant NP, defective in NP-NP interaction, exhibited a dominant negative phenotype in the MG assay (Figure 7) and interfered with virus multiplication (Figure 8). This might be explained because NP (D471G) can still bind RNA, and it could compete with wt NP for the RNA substrate during the initial phase of viral RNA encapsidation. Likewise, NP (D471G) is likely to interact with L [61] and compete with NP (wt)-L interaction required for the formation of the functional virus polymerase complex. Moreover, NP (D471G) has the ability to interact with the Z protein and thereby compete with the Z-vRNP interaction required for production of matured infectious virions. It should be noted that for a variety of NS RNA viruses, mutations affecting NP-NP interaction have been documented to exhibit dominant negative phenotypes [62]. These findings support the feasibility of targeting NP-NP interaction as a novel antiviral strategy to combat arenavirus infections
